# Effects of Complex Movement on the Excitability of the Ipsilateral Primary Motor Cortex and Spinal Motoneurons Contralateral to the Movement: A Comparison of Ball Rotation and Grasping Tasks with Equivalent Muscle Activity

**DOI:** 10.3390/brainsci15020171

**Published:** 2025-02-10

**Authors:** Rin Kosuge, Takehiro Sukegawa, Mayu Akaiwa, Eriko Shibata, Ryo Kurokawa, Yasushi Sugawara, Satoshi Kudoh, Yuya Matsuda, Hidekazu Saito, Takeshi Sasaki, Kazuhiro Sugawara

**Affiliations:** 1 Graduate School of Health Sciences, Sapporo Medical University, Sapporo 060-8556, Japan; 2 Physical Therapy Division, Department of Rehabilitation, Hokuto Social Medical Corporation Tokachi Rehabilitation Center, Obihiro 080-0833, Japan; 3 Department of Rehabilitation, Kashiwaba Neurosurgical Hospital, Sapporo 062-0051, Japan; 4 Major of Physical Therapy, Department of Rehabilitation, Faculty of Healthcare and Science, Hokkaido Bunkyo University, Eniwa 061-1449, Japan; 5 Department of Occupational Therapy, School of Health Sciences, Sapporo Medical University, Sapporo 060-8556, Japan; 6 Department of Physical Therapy, School of Health Sciences, Sapporo Medical University, Sapporo 060-8556, Japan

**Keywords:** transcranial magnetic stimulation, ipsilateral corticospinal tract, complex movement, electromyography, spinal motoneurons

## Abstract

**Background/Objectives:** Unilateral hand movements alter the excitability of the ipsilateral primary motor cortex (ipsi-M1) and contralateral spinal motoneurons. Although this excitability increases during complex, high muscle-activity movements, few studies have examined the excitability of ipsi-M1 and contralateral spinal motoneurons during complex movements while accounting for muscle activity. This study investigated the excitability of ipsi-M1 and contralateral spinal motoneurons during complex and simple movement tasks with comparable muscle activity between the two tasks. **Methods:** Nineteen healthy adult volunteers participated in this study. The ball rotation task was set as the complex movement task (BR condition), and the grasping task was set as the simple movement task (grasp condition), with peak muscle activity values comparable between the tasks. Motor-evoked potentials (MEPs) and F-waves were recorded from the abductor pollicis brevis muscle contralateral to the movement during task execution. The excitability parameters of ipsi-M1 and contralateral spinal motoneurons were calculated by dividing the MEP, F-wave persistence, and F/M amplitude values recorded in each condition by the corresponding values recorded at rest. These parameters were compared across the rest, BR, and grasp conditions. **Results:** All the excitability parameters of ipsi-M1 and contralateral spinal motoneurons increased during both the BR and grasp conditions compared with the rest condition but did not differ significantly between the BR and grasp conditions. **Conclusions:** The excitability of ipsi-M1 and contralateral spinal motoneurons was strongly influenced by the amount of muscle activity but not by the complexity of the movement.

## 1. Introduction

The excitability of the contralateral corticospinal tract in the control of unimanual hand movements increases during voluntary movement of the unilateral hand [1,2,3,4,5]. Several studies on transcranial magnetic stimulation (TMS) have shown that the excitability of the primary motor cortex (M1) ipsilateral to the movement (ipsi-M1) and the M1 contralateral to the movement (contra-M1) increases during voluntary movement of the unilateral hand [6,7,8,9,10]. The increased excitability of ipsi-M1 is greater during complex movements that require dexterity than during simple movements [7,9]. The mechanism underlying the increased ipsi-M1 excitability may involve an increase in interhemispheric inhibition from contra-M1, which reduces the intracortical inhibition of ipsi-M1 and releases inhibitory influences on corticospinal output neurons [11,12]. Similarly, in patients with stroke, the excitability of ipsi-M1 is increased by movement of the affected side, the extent of which is greater than that in healthy participants [13]. Although the increased excitability of ipsi-M1 during the use of the affected side in patients with stroke potentially contributes to recovery after stroke [13], some reports have associated this increase with poor recovery [14,15]. Thus, the mechanism and functional role of the excitability of ipsi-M1 in patients with stroke remain unclear. The excitability of ipsi-M1 may also be influenced by complex movements in patients with stroke [16], which could partially explain the similarities in ipsi-M1 excitability between these patients and healthy participants. Understanding the excitability of ipsi-M1 would help establish an effective treatment program for patients with stroke. Therefore, knowledge of the excitability of ipsi-M1 is essential [16].

The corticospinal tract is a nerve conduction pathway that descends from the M1 to the spinal motoneurons. Taniguchi et al. [17] found that prolonged immobility reduces the excitability of contra-M1 and spinal motoneurons ipsilateral to the movement, whereas voluntary contractions restore it. Rothwell et al. [18] also reported that TMS to contra-M1 increased the excitability of contralateral spinal motoneurons. These reports demonstrate that the upper motor neurons in M1 facilitate the excitability of spinal motoneurons. Therefore, during complex movements, the excitability of these neurons is increased not only in ipsi-M1 but also in contralateral spinal motoneurons. However, few studies have investigated the excitability of contralateral spinal motoneurons during complex movements.

The excitability of ipsi-M1 may increase during isometric exercises involving the strong contraction of the unilateral hand [19,20,21]. Similarly, during isometric exercises in the unilateral hand, the excitability of contralateral spinal motoneurons can increase with muscle contraction strength [20,21]. The excitability of ipsi-M1 and contralateral spinal motoneurons is influenced by muscle contraction strength, and the effect of muscle activity should be considered when investigating these factors in complex movements. However, few studies have investigated the excitability of ipsi-M1 and contralateral spinal motoneurons while measuring muscle activity on the performance side during movement tasks. This study investigated the excitability of ipsi-M1 and contralateral spinal motoneurons during simple and complex movement tasks with comparable muscle activity using motor-evoked potentials (MEP) and F-waves evoked from the contralateral homologous muscle. This study hypothesized that the excitability of ipsi-M1 and contralateral spinal motoneurons would be significantly increased during the complex task compared with the simple task.

## 2. Materials and Methods

### 2.1. Participants

We recruited 19 healthy young adults (mean ± standard deviation age: 23.5 ± 1.5 years; 12 male, 7 female). Exclusion criteria included a history of epilepsy, other neurological conditions, medical conditions that could affect muscle function, such as hand injuries, and being a musician. Musicians were defined as individuals with at least 9 years of experience playing a musical instrument and practicing at least 5 h per week over the past 5 years [22]. All participants were confirmed not to meet any of the exclusion criteria and were verified to be right-handed using the Flinders Handedness Questionnaire [23]. All participants provided written informed consent. The study was approved by the Ethics Committee of Sapporo Medical University (approval no. 2-1-91) and conducted in accordance with the 1964 Declaration of Helsinki and its later amendments or comparable ethical standards.

### 2.2. Study Design

**Figure 1:**
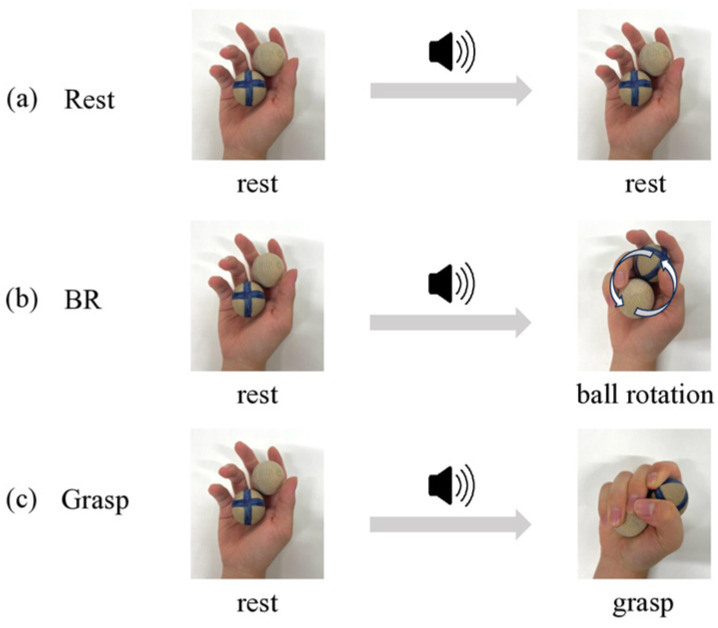
Three movement conditions under which the excitability of ipsi-M1 and contralateral spinal motoneurons was measured. In the rest condition (**a**), two wooden balls were placed in the right palm, but the hand remained at rest. In the ball rotation (BR) condition (**b**), following the sound stimulus, two wooden balls were held in the right palm and rotated counterclockwise. In the grasp condition (**c**), following the sound stimulus, two wooden balls were grasped with the right hand.

The participants were seated in a comfortable chair with their bilateral upper limbs resting on a table on either side of the chair. Two wooden balls were placed on the right palm, and the excitability of ipsi-M1 and contralateral spinal motoneurons was measured under the following three conditions (Figure 1): (1) rest condition, in which two wooden balls were placed on the right palm and the hand was kept at rest; (2) ball rotation (BR) condition, in which the two wooden balls were rotated counterclockwise on the right palm as the complex movement task [24,25]; and (3) grasp condition, in which the two wooden balls were grasped with the right hand as the simple movement task. The last two tasks both involved equivalent controlled muscle activity of the abductor pollicis brevis (APB). In the BR and grasp conditions, participants were instructed to perform one movement after each sound stimulus (electronic tones). In total, 40 sound stimuli were created every 5000 ms in both the BR and grasp conditions. TMS or electrical stimulation was applied 700 ms after the sound stimuli. TMS was applied 15 times and electrical stimulation 25 times in a random order. The experiment was performed over 2 days. On day 1, the rest and BR conditions were performed in random order. On day 2, the rest and grasp conditions were also performed in random order. To ensure that the electromyography (EMG) levels were equal between the BR and grasp conditions, the normalized peak EMG was calculated for each participant during the BR condition. The normalized peak EMG was calculated by dividing the average of the peak values (total: 40 peak values) of the rectified EMG recorded 5000 ms after each sound stimulus in the BR condition by the 100% EMG value. This value was then used as a guideline for contraction strength in the grasp condition. In the grasp condition, the normalized peak EMG was displayed on the monitor in front of the participants. The experimental session in the grasp condition was performed after the participants had practiced aligning their peak EMG with the normalized peak EMG during grasping movements.

### 2.3. EMG Measurement

EMG was measured using an Ag/AgCl electrode (BlueSensor NF-00; Ambu, Ballerup, Denmark) mounted above the bilateral APB. EMG signals (DL-140; 4 Assist, Tokyo, Japan) were sampled at 10 kHz (Power Lab; AD Instruments, Dunedin, New Zealand) and band-pass filtered at 0.1–500.0 Hz. The EMG signals were rectified and smoothed using a digital filter with 101 points. Before the measurements conducted on days 1 and 2, maximum voluntary contraction of the palmar abduction of the right thumb was performed for 5 s. The mean value of the measurement at 3 s, excluding 1 s before and after, was calculated as 100% EMG.

### 2.4. MEP Measurement

MEP was used to assess the excitability of ipsi-M1 (i.e., right hemisphere-M1). TMS was applied using a figure-eight-shaped coil (diameter: 95 mm) connected to a stimulator (Magstim 200; Magstim, Whitland, UK). The coil was placed at the optimal position (hot spot) for eliciting MEPs [26]. The coil handle was positioned backward at a 45° angle to the midsagittal line [7,27]. Participants wore swimming caps, and the outer edge of the coil was marked with a pen to stabilize its position during the experiment. The resting motor threshold (rMT) of the left APB was determined as the minimum stimulus intensity required to evoke an MEP amplitude of at least 50 µV in the resting APB muscle in five out of ten trials [26]. The TMS intensity was set at 120% of rMT [7].

### 2.5. F-Wave Measurement

F-waves were used to assess the excitability of contralateral spinal motoneurons. An evoked potential testing device (Neuropack MEB-2312, Nihon Kohden, Tokyo, Japan) was used to record F-waves. Electrical stimuli were applied to the left median nerve for 0.2 ms at the left wrist, with a stimulus intensity set to 120% of the stimulation intensity required to elicit maximum M-waves (Mmax) [28].

### 2.6. Data Analysis

In the BR and grasp conditions, the mean EMG activity from the right APB measured during task performance was calculated and normalized to 100% EMG. The MEP amplitude was calculated from the peak-to-peak amplitudes of 13 trials, excluding the maximum and minimum MEP amplitudes [29]. Trials in which the rectified EMG activity of the left APB exceeded 0.02 mV during the 50 ms before the TMS pulse were excluded [30]. To compare changes in ipsi-M1 excitability from the rest condition between the BR and grasp conditions, the MEP ratio (MEP amplitude in the three conditions/MEP amplitude in the rest condition) was calculated. F-waves with peak-to-peak values of ≥50 µV were included in the analysis [31]. The F-wave persistence (FP) and F/M amplitude (F/M-amp) ratio were used as parameters of the excitability of the left-side spinal motoneurons (i.e., spinal motoneurons contralateral to the movement). The FP was defined as the percentage of F-wave occurrences relative to the number of stimuli. The F/M–amp ratio was defined as the percentage of the mean F-wave amplitude relative to Mmax. To compare the differences in each of the two parameters under the BR and grasp conditions with those under the rest condition, the FP variation (FP in the three conditions/FP in the rest condition) and F/M–amp variation (F/M–amp ratio in the three conditions/F/M–amp ratio in the rest condition) were calculated.

### 2.7. Statistical Analysis

The Shapiro–Wilk test was performed, which revealed a non-normal distribution of the data. The %EMG between the BR and grasp conditions was compared using the non-parametric Wilcoxon signed-rank test, with the significance level set at *p* < 0.05. The non-parametric Friedman test was then used to compare differences in each excitability parameter (MEP ratio, FP variation, and F/M-amp variation) between the BR and grasp conditions and the rest condition. Statistical significance for the Friedman test was defined as *p* < 0.05. If the Friedman test indicated a significant difference, the non-parametric Wilcoxon signed-rank test with Bonferroni correction was performed, with statistical significance defined as *p* < 0.05/3 = 0.016. Statistical analyses were conducted using IBM SPSS 25 software (IBM Corp., Armonk, NY, USA).

## 3. Results

### 3.1. EMG Activity During Movement Tasks

**Figure 2:**
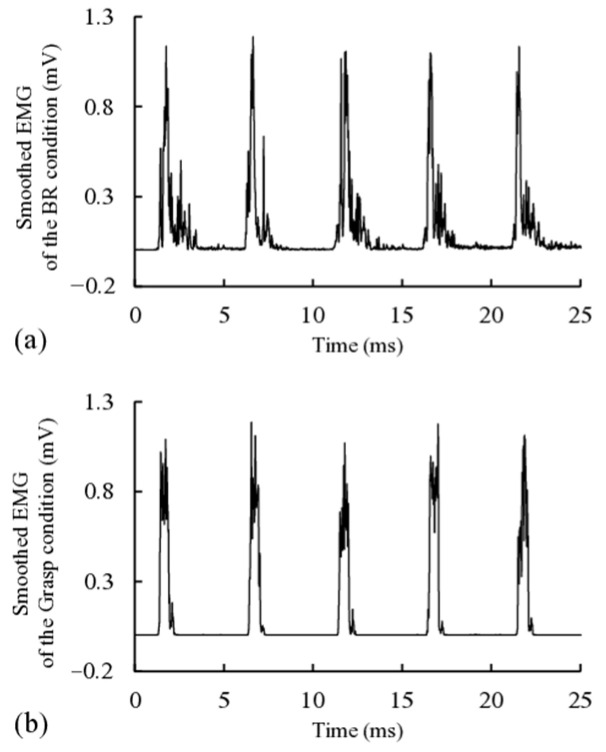
Smoothed electromyography waveforms recorded from the right abductor pollicis brevis of a representative participant in the BR (**a**) and grasp (**b**) conditions.

**Figure 3:**
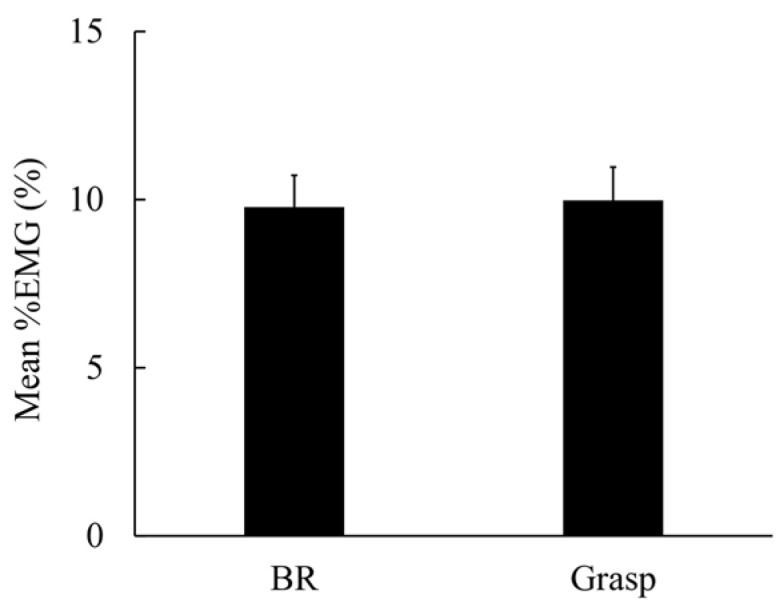
Mean electromyography percentage during the performance of the BR and grasp conditions. Error bars represent the standard error of the mean.

Figure 2 shows a representative image of smoothed EMG waveforms recorded from the right APB of a participant under the BR and grasp conditions. In the BR condition, the EMG amplitude peaked and then gradually declined to baseline (Figure 2a). The EMG waveform for the grasp condition was characterized by a shorter time from baseline to peak EMG amplitude and a shorter time from peak to baseline compared with the BR condition (Figure 2b). Figure 3 shows the mean %EMG during the performance of the BR and grasp conditions. The mean ± standard error of the mean %EMG was 9.78% ± 0.94% in the BR condition and 9.98% ± 0.99% in the grasp condition. No significant difference was found in %EMG between the BR and grasp conditions (*p* = 0.809).

### 3.2. MEP Ratio

**Figure 4:**
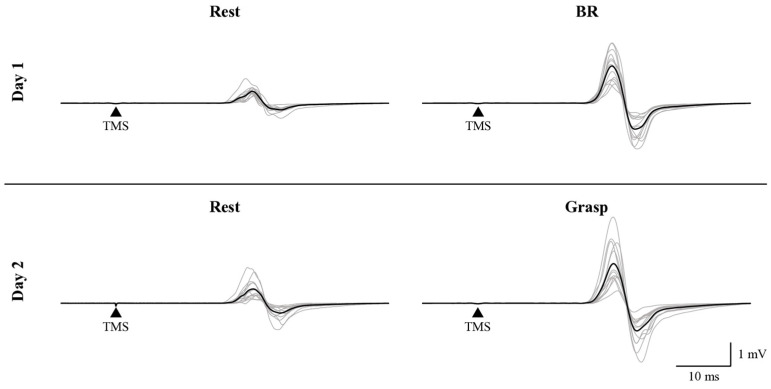
Representative motor evoked potential (MEP) waveforms for the left abductor pollicis brevis of a participant in the rest, BR, and grasp conditions. The black line represents the average MEP waveform for each condition, whereas the gray lines represent the MEP waveforms for individual trials.

**Figure 5:**
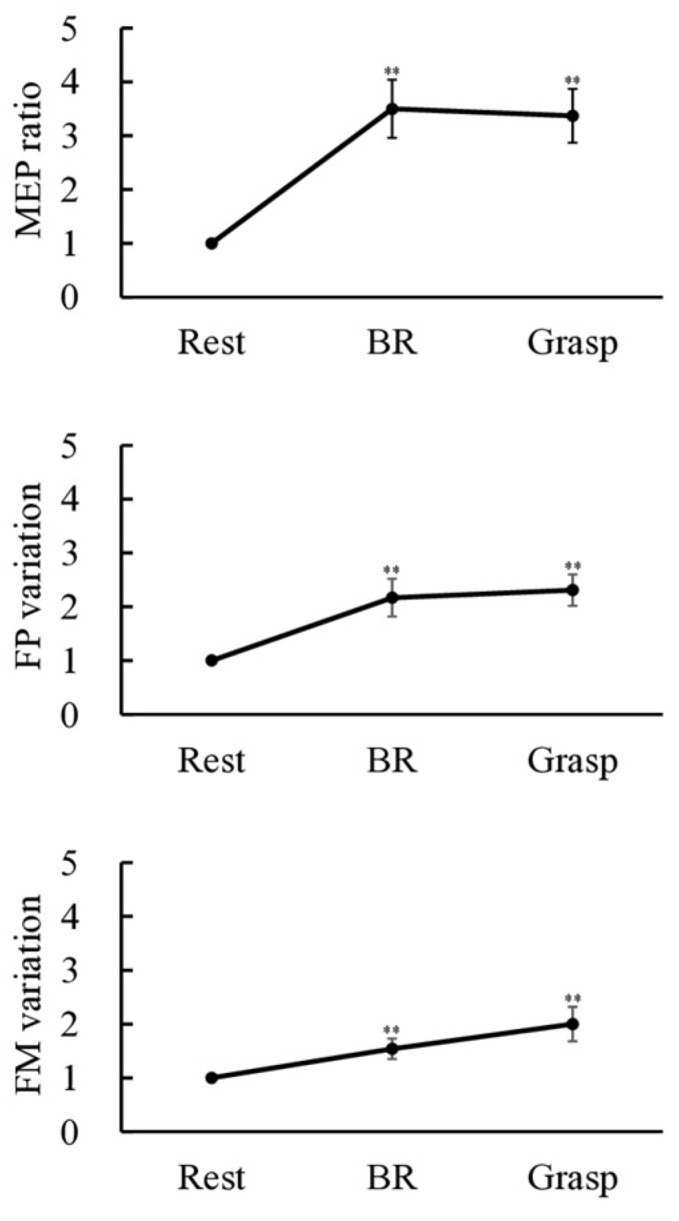
Excitability parameters for each of the three conditions. Error bars represent the standard error of the mean, and asterisks (**) indicate significant differences (*p* < 0.01) compared with the rest condition.

Figure 4 shows typical waveforms of MEP under the three conditions (rest, BR, and grasp). Figure 5 and Table 1 present the results of the MEP ratio (normalized to “1” for the rest condition) under the three conditions. The Friedman test revealed significant differences in the MEP ratio (χ^2^ (2) = 21.158, *p* < 0.001). The Wilcoxon signed-rank test showed a significant increase in the MEP ratio for both the BR and grasp conditions compared with the rest condition (BR condition, *p* < 0.001; grasp condition, *p* < 0.001). Conversely, no significant differences were observed in the MEP ratio between the BR and grasp conditions (*p* = 0.243).

### 3.3. FP Variation/FM–Amp Variation

**Figure 6:**
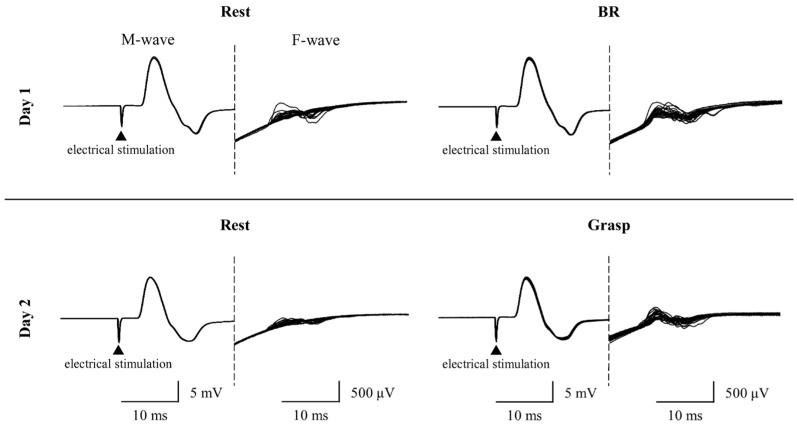
Representative F-wave waveforms for the left abductor pollicis brevis of a participant in the rest, BR, and grasp conditions.

Figure 6 shows typical waveforms of F-wave under the three conditions (rest, BR, and grasp). Figure 5 and Table 1 present the results of FP variation and F/M–amp variation (normalized to “1” for the rest condition) under the three conditions. The Friedman test revealed significant differences in both FP variation and F/M-amp variation (FP variation: χ^2^ (2) = 23.625, *p* < 0.001; F/M-amp variation: χ^2^ (2) = 14.000, *p* < 0.001). The Wilcoxon signed-rank test showed a significant increase in FP variation and F/M–amp variation for the BR and grasp conditions compared with the rest condition (FP variation: BR condition, *p* = 0.001; grasp condition, *p* < 0.001; F/M–amp variation: BR condition, *p* = 0.009; grasp condition, *p* = 0.001). Conversely, no significant differences were observed in FP variation or F/M–amp variation between the BR and grasp conditions (FP variation: *p* = 0.407; F/M–amp variation: *p* = 0.147).

## 4. Discussion

We compared the excitability of ipsi-M1 and contralateral spinal motoneurons during complex (BR) and simple (grasp) movements of the unilateral hand. The excitability parameters showed a significant increase during both the BR and grasp movements compared with the rest condition. However, no significant differences were observed in any of the excitability parameters between the BR and grasp movements.

In this study, the excitability of ipsi-M1 and contralateral spinal motoneurons increased during the two movement tasks compared with the rest condition. Although the mechanism underlying the increased excitability of ipsi-M1 with increased muscle contraction strength remains unclear, Andrushko et al. [19] reported a positive correlation between the grasp strength of the unilateral hand and changes in ipsi-M1 excitability. Studies suggest that the excitability of ipsi-M1 supports contra-M1 and enhances the output required for grasping [19,32]. Additionally, the increased excitability of ipsi-M1 may contribute to the increased excitability of spinal motoneurons contralateral to the movement [17,18]. Thus, the excitability of ipsi-M1 and contralateral spinal motoneurons increased during the BR and grasp tasks due to the muscle contractions of the unilateral hand. However, previous studies have shown that isometric contractions of the unilateral hand during low-level muscle activity do not alter the excitability of contralateral spinal motoneurons [10,33]. In the present study, the mean muscle activity during movement was at a low level of approximately 10% EMG in both the BR and grasp conditions, yet the excitability of ipsi-M1 and contralateral spinal motoneurons still increased. One possible explanation for this discrepancy is that the movement task in the present study involved isotonic rather than isometric contractions. Johannsen et al. [34] reported that the excitability of M1, the supplementary motor cortex, and the premotor cortex increased during isotonic contractions compared with isometric contractions. Similarly, Yahagi et al. [35] demonstrated that corticospinal excitability increased during isotonic contractions compared with isometric contractions. Based on these findings, the isotonic contraction tasks implemented in the present study may have increased the excitability of contralateral spinal motoneurons by enhancing the excitability of ipsi-M1, even with low levels of muscle activity.

In this study, no significant differences were found in the excitability of ipsi-M1 and contralateral spinal motoneurons during complex and simple movements with comparable muscle activity. These results were inconsistent with those of previous studies [3,4], which reported increased excitability of ipsi-M1 in complex movement tasks compared with simple movement tasks. However, Tinazzi and Zanette [9] did not measure muscle activity, and this inconsistency could be explained by differences in muscle activity between tasks. Conversely, Morishita et al. [7] measured muscle activity in the task-performing hand. Their complex movement task involved picking up a glass ball with chopsticks and transferring it between boxes, whereas their simple movement task consisted of repetitive grasping movements using the thumb, index, and middle fingers at a frequency of 1 Hz. They found that activity in the first dorsal interosseous (FDI) muscle of the task-performing hand was not related to the MEP amplitude in the resting FDI. However, because their study involved an experimental setup with the elbow fixed, the complex movement task likely engaged the elbow flexors and shoulder joint internal/external rotators in addition to the hand muscles. Conversely, the involvement of muscles beyond the hand muscles was unlikely in the present study, as both tasks were confined to the joint movement of the hand. Several previous studies have demonstrated that ipsi-M1 excitability increases with strong contraction of the unilateral limb [19,32] and with the activation of non-homologous muscles [33,36], suggesting that ipsi-M1 excitability is influenced by muscles other than the hand muscles on the active side. Therefore, the differences in ipsi-M1 excitability observed by Morishita et al. may be attributed to increased muscle activity in both the hand and upper limb during the complex movement task compared with the simple task. For these reasons, the present findings, which showed no differences in the excitability of ipsi-M1 and contralateral spinal motoneurons between complex and simple movements, indicate that the excitability of ipsi-M1 and contralateral spinal motoneurons may be strongly influenced by muscle activity rather than by the complexity of the movement task.

Previous studies have reported that bilateral M1 excitability increases when patients with stroke move the affected hand [37]. Although the increased excitability of ipsi-M1 during use of the affected hand contributes to recovery after stroke [13], some studies have associated this increased excitability with poor recovery outcomes [14,15]. Thus, although no consensus has been reached regarding the relationship between ipsi-M1 excitability and functional recovery, these studies consistently highlight the importance of changes in ipsi-M1 excitability for functional recovery in patients with stroke. In the present study, the excitability of ipsi-M1 increased regardless of movement complexity under similar levels of muscle activity. Therefore, when aiming to modulate cortical excitability in stroke rehabilitation, the focus should be on evaluating muscle activity rather than task complexity. However, as this study included only healthy adults, future research should investigate whether similar results are observed in patients with stroke.

This study has three limitations. First, the activity of brain regions other than ipsi-M1 during each condition was not investigated, as only TMS was used to assess ipsi-M1 excitability. The BR task, designed as a complex movement task, involves continuous movements and activates multiple motor-related regions [25]. However, in this study, we focused on investigating excitability changes in ipsi-M1 to the movement using TMS. Consequently, the role of contra-M1 in movement tasks remains uncertain, as well as the contributions of other motor-related regions involved in motor control. Moreover, the neural mechanism responsible for the increased excitability of ipsi-M1 is thought to involve interhemispheric inhibition between homologous cortical areas mediated by the corpus callosum, resulting in a reduction of short-interval intracortical inhibition in ipsi-M1 [8,38]. Indeed, studies using fMRI and PET to measure the brain activities during complex motor tasks have reported the activation not only in contra-M1 but also in ipsi-M1 [25,39,40]. Future studies should therefore employ MRI to comprehensively investigate the brain regions functionally connected to M1, which are hypothesized to contribute to complex finger movements. Second, although the stimuli were applied during the movement task, the movement phases could not be perfectly aligned, as the time required for one BR varied between individuals. Because the contraction strength of muscles involved in BR movement, such as the APB, flexor digitorum superficialis, and flexor digitorum profundus, may differ across movement phases, variations in muscle contraction strength might have influenced the excitability of ipsi-M1 and contralateral spinal motoneurons. Third, the BR condition was always performed on day 1 for all participants, and it is possible that TMS and/or familiarity with the experimental protocol influenced excitability.

## 5. Conclusions

In the present study, we investigated the excitability of ipsi-M1 and contralateral spinal motoneurons during simple and complex movement tasks with comparable muscle activity. Our results demonstrated that the complexity of the movement task did not influence the excitability of ipsi-M1 and contralateral spinal motoneurons, suggesting that muscle activity is the primary factor contributing to the observed increases in excitability.

## Figures and Tables

**Table 1:** Excitability parameters in the BR and grasp conditions (mean ± standard error of the mean).

Parameter	BR	Grasp
MEP ratio	3.50 ± 0.54	3.37 ± 0.50
FP variation	2.17 ± 0.35	2.31 ± 0.29
F/M-amp variation	1.54 ± 0.19	2.00 ± 0.32

MEP ratio, motor-evoked potential (MEP) amplitude for the three conditions divided by the MEP amplitude in the rest condition; FP variation, F-wave persistence (FP) in the three conditions divided by FP in the rest condition; F/M–amp variation, F/M–amplitude (F/M–amp) ratio in the three conditions divided by the F/M–amp ratio in the rest condition.

## Data Availability

The data presented in this study are available on request from the corresponding author due to privacy reasons.
